# Spinal Cord Injury Remyelination: Pathways to Therapies

**DOI:** 10.3390/ijms26157249

**Published:** 2025-07-26

**Authors:** Julia K. Kaniuk, Divy Kumar, Joshua Tennyson, Kaitlyn L. Hurka, Alexander Margolis, Andrei Bucaloiu, Ashley Selner, Christopher S. Ahuja

**Affiliations:** 1 Feinberg School of Medicine, Northwestern University, 240 E Huron Street, Suite 1-200, Chicago, IL 60611, USA; julia.kaniuk@northwestern.edu (J.K.K.); divy.kumar@northwestern.edu (D.K.); joshua.tennyson@northwestern.edu (J.T.); kaitlyn.hurka@northwestern.edu (K.L.H.); alex.margolis@northwestern.edu (A.M.); andrei.bucaloiu@northwestern.edu (A.B.); 2 Northwestern Medicine Department of Neurological Surgery, 676 N St Clair Street, Suite 2210, Chicago, IL 60611, USA; ashley.selner@nm.org; 3 Simpson-Querrey Research Institute, 303 E. Superior St., Chicago, IL 60611, USA

**Keywords:** spinal cord injury, neuronal degeneration, remyelination, myelin repair, neuroinflammation, stem cell therapy, stem cells, neuroregeneration, neuroplasticity, molecular changes

## Abstract

Spinal cord injury (SCI) is a debilitating condition that results from a culmination of acute and chronic damage to neural tissue, specifically the myelin sheath, thus impacting neurons’ abilities to synergistically perform their physiological roles. This review explores the molecular underpinnings of myelination, demyelination, and remyelination, emphasizing the role of oligodendrocyte progenitor cells (OPCs), astrocytes, and microglia in physiological, and pathophysiological, healing. Furthermore, we link these processes with emerging therapeutic strategies currently under investigation in animal and human models, underscoring areas of translational medicine that remain underutilized. The goal of this review is to provide a framework for developing more advanced interventions to restore function and improve outcomes for individuals with SCI.

## 1. Introduction

Myelin is a lipid-rich sheath, composed of cholesterol, phospholipids, galactolipids, and plasmalogens that envelops and insulates axons, enabling fast and efficient nerve signal transmission [1]. The oligodendrocyte (OL) is the primary glial cell synthesizing, compacting, and maintaining myelin in the central nervous system (CNS), with each OL capable of myelinating multiple axonal internodes [1]. Myelin sheaths are not continuous down the axon but rather consist of myelinated internodes punctuated by small regularly spaced, unmyelinated gaps known as nodes of Ranvier where voltage-gated sodium channels are clustered for action potentials to occur and sustain the nerve signal impulses [2,3]. This saltatory conduction in myelinated axons is significantly higher in velocity (up to 150 m/s) than conduction in unmyelinated axons (0.5 to 10 m/s) [4]. In addition to supporting efficient impulse conduction, myelin also provides metabolic support to axons through its connection to astrocytes and OLs that process lactate and other trophic factors that provide both ATP and carbon skeletons for lipid production [5]. Myelination is necessary for efficient and effective nerve signal conduction and metabolic support of the axon, and its absence, dysfunction, or destruction can cause serious functional impairments.

Spinal cord injury (SCI) leads to a breakdown of myelin and release of cellular debris that sets off an inflammatory cascade amplified by reactive oxygen species (ROS) and pro-inflammatory cytokines that promote axon retraction and dieback. Macrophages and complement interact with myelin to clear debris, ultimately reduce inflammatory processes, and set the stage for neural repair [6]. OPCs, multipotent adult CNS stem cells, become activated in settings of demyelinating damage, differentiate into OLs, and together with astrocytes, lead and support the nascent myelination process, respectively [6]. Therapies aiming to stimulate OPCs including cell transplantation, pharmacological therapies, and molecular pathway interventions can effectively stimulate OPC proliferation and differentiation, improving myelin sheath formation in both in vitro and in vivo models. Approaches that integrate neuroprotection, axonal regeneration, and remyelination are areas of current research aiming to optimize therapeutic benefits [7].

While demyelination of axonal segments results in impaired conduction and block, even partial remyelination might be sufficient to regain lost function and foster a more supportive environment for neural repair [8,9]. Restoring the myelin sheath mitigates the persistence of chronic inflammation, reducing tissue damage [10]. Remyelination also stabilizes axons and supports the formation of new synapses, enabling functional compensation as connections are lost and made throughout life; myelin plasticity aids in re-establishing motor and sensory circuits post-injury and is also theorized to play a role in motor-spatial learning and working memory [11]. Yet, when the clearance of debris, the inflammatory cascade, and the remyelination process goes awry, chronic inflammation and demyelination persist, leading to poor outcomes.

This is an active area of research and therapeutic innovation. In pediatric patients with multiple sclerosis (MS), a progressive demyelinating disease, it has been shown that lower physical activity levels increase the lesion burden [12]. In adult patients with MS, it has been shown that exercise enhances the immune cell-mediated debris clearance within lesions and augments OPC proliferation and the rate of remyelination [12]. Combining physical therapy and rehabilitation with targeted molecular remyelination therapies could bolster myelin debris clearance, enhance remyelination, and greatly improve outcomes for patients suffering from SCI. This review will explore the biological underpinnings of remyelination, outline existing pharmacological and non-pharmacological therapies for SCI, and comment on future research directions crucial to improving patient outcomes.

## 2. Pathology of Myelination in SCI

### 2.1. Impact of SCI on Myelin

#### 2.1.1. Primary Injury

SCI is divided into two stages of injury, primary and secondary. Primary injury describes direct mechanical trauma to the spinal cord (SC) and can be described with one of four potential mechanistic models of injury: (1) impact plus persistent compression, (2) impact plus transient compression, (3) distraction (stretching in the axial plane), and (4) laceration/transection [13]. The mechanical forces involved in primary SCI cause direct physical damage to axons, myelin, OLs, and microcirculation [14]. Primary SCI also causes systemic effects including neurogenic shock and subsequent bradycardia and hypotension that can further contribute to worsening ischemia of the SC if left untreated [13,14]. These systemic effects compound with the local injury to exacerbate SC degeneration.

Axon–myelin interactions are disrupted by initial mechanical impact during primary injury through damage to the paranodal myelin region, the region flanking nodes of Ranvier where OLs interact closely with axons [15]. Damage to the paranodal region results in exposure and redistribution of potassium channels in the juxtaparanodal region that are normally covered by the myelin sheath [16]. Axons also provide trophic support to myelin, so interruption of the axon–myelin interaction or neuronal cell death during primary injury may contribute to demyelination [17]. The initial insult leads to immediate OL death via necrosis and apoptosis, resulting in impaired saltatory conduction, conduction block, and rapid loss of action potential fidelity, which are key contributors to early neurological deficits. This demyelination not only impairs axonal conduction but also renders axons more vulnerable to subsequent degeneration and secondary injury cascades [18,19]. Furthermore, OPCs constitute 5–8% of CNS cells, and thus are directly affected by primary injury. OPCs are highly proliferative and widely distributed, allowing for rapid response to injury [20]. In the acute phase, many OPCs in the lesion core are lost due to mechanical and excitotoxic injury, while those in the perilesional area proliferate in response to injury signals [21].

The immediate depletion of both OLs and OPCs during primary injury limits the endogenous capacity for remyelination in the acute phase. The hostile post-injury environment, characterized by inflammation, oxidative stress, and inhibitory extracellular matrix molecules, further impairs OPC differentiation [20,22]. Although OPCs are capable of mounting a proliferative response to injury, their remyelination capacity is significantly restricted in the context of acute injury. This acute OL loss is a critical determinant of the extent of demyelination and sets the stage for the secondary injury phase, where the potential for endogenous remyelination by surviving OPCs becomes a key factor in long-term recovery [23,24].

#### 2.1.2. Secondary Injury

Secondary injury involves the complex cascade of downstream events following primary injury that further contribute to SC degeneration [13,25]. Beyond the initial insult to microvasculature during primary injury, there are further changes in microcirculation including hemorrhage, vasospasm, thrombosis, and impaired autoregulation that compromise perfusion to the site of injury [14]. This lack of perfusion and resulting ischemia extends rostrally and caudally from the site of injury and threatens the survival of OLs. OLs are highly sensitive to oxygen and nutrient deprivation. Ischemia rapidly induces their death via necrosis and apoptosis, resulting in the demyelination of spared axons and loss of axonal conduction integrity [26,27]. OPCs, while capable of proliferating and migrating toward the lesion, are also susceptible to the hostile ischemic microenvironment. Hypoxia and metabolic stress impair OPC survival, limit their differentiation into mature OLs, and reduce their capacity to remyelinate denuded axons. The combination of OL loss and impaired OPC function leads to insufficient remyelination in the subacute and chronic phases after injury [21]. Interestingly enough, only about 5–10% of the original axons, particularly in the nonpyramidal tract, must persist for residual neurological function to remain after SCI [28].

Excitotoxicity also contributes to secondary injury. Excess glutamate release leads to immediate neuronal cell death and delayed white matter degeneration through excitotoxic mechanisms targeting both OLs and OPCs [25]. OLs and OPCs express both AMPA and NMDA receptors, which, when activated by high extracellular glutamate, permit excessive influx of calcium ions into these cells. This calcium overload triggers mitochondrial dysfunction, activation of apoptotic pathways, and ultimately resulting in apoptosis in OLs [29].

AMPA receptor-mediated excitotoxicity is particularly prominent in OLs, as these receptors are highly permeable to calcium in the absence of the GluR2 subunit. Activation of these receptors leads to sustained intracellular calcium elevation, mitochondrial cytochrome c release, and cell death. NMDA receptors, which are also present on OLs and their processes, contribute to calcium influx and are activated under ischemic and traumatic conditions, further exacerbating OL loss and demyelination [30]. One study demonstrated that antagonism of NMDA receptors and subsequent blockage of glutamate activity in rats was associated with improved functional recovery following SCI [31]. Thus, glutamate excitotoxicity plays a key role in secondary injury after SCI.

Free radical production following SCI is another avenue of secondary injury. Increased levels of free radicals following SCI have been demonstrated through elevated levels of malondialdehyde, a final product of free radicals which can be more reliably measured than short-lived free radicals [32]. Following SCI, mitochondrial stress and subsequent calcium overload activates nitrogen oxidase which generates reactive nitrogen species (RNS) and induces generation of superoxide by the electron transport chain [33]. Oxidation of Fe^2+^ to Fe^3+^ following the release of iron from intracellular ferritin gives rise to more ROS [34]. ROS and RNS directly oxidize lipids, proteins, and DNA within OLs and OPCs, leading to membrane disruption, myelin sheath breakdown, and activation of apoptotic pathways. OLs are particularly vulnerable due to their high metabolic demand, iron content, and relatively low antioxidant capacity, making them a primary target for oxidative injury. OPCs also exhibit impaired survival and differentiation under oxidative stress, further limiting their endogenous post-injury repair [23,35,36,37]. Targeting oxidative stress pathways, such as inhibiting NADPH oxidase or enhancing antioxidant defenses, has been shown to reduce OL loss and improve outcomes in preclinical models.

Epigenetic histone modifications are commonly observed after SCI [35,38]. Modifications such as those mediated by the polycomb repressive complex 2, can repress or de-silence key genes required for OPC differentiation and OL maturation. After SCI, acute upregulation of repressive operons and changes in chromatin accessibility disrupt the transcriptional landscape of OLs, leading to the downregulation of myelin-related genes and impaired myelin sheath maintenance [24,39]. Transcriptional dysregulation of several regulatory myelination genes, such as Plp1, Hexa, and Hexb further impairs the ability of OPCs to differentiate into mature, myelinating OLs, and compromises the structural and metabolic support provided to axons [40]. Previous work has also shown that some altered microRNA levels, such as miR-219, can negatively impact remyelination. MiR-219 is a key regulator of the transition from OPC to mature OL, and its downregulation leads to persistent pools of undifferentiated OPCs and insufficient generation of new myelinating OLs [21].

Inflammation following SCI has both beneficial and deleterious effects [41]. Various immune cells including neutrophils, resident macroglia and microglia, dendritic cells, bloodborne macrophages, and B and T lymphocytes are recruited to the site of injury following SCI. Some of these cells such as neutrophils, microglia, and macrophages are beneficial and necessary for the clearance of cell debris and damaged tissue [34]. However, lymphocytes reactive to myelin basic protein (MBP) are present in increased levels following SCI [42]. These autoreactive lymphocytes exacerbate and prolong inflammation, with autoreactive T cells being directly toxic to neurons and OLs and can also target OPCs [41]. IFN-γ and other pro-inflammatory cytokines from T cells and activated astrocytes suppress OPC differentiation and promote OPC death, compounding the failure of remyelination [43]. One study found that athymic rats had greater tissue preservation and functional recovery following SCI compared to rats with functional T cells, demonstrating the damaging effects of autoreactive T cells following SCI [44].

While the precise role of B cells following SCI is unclear, evidence suggests that B cells mediate a chronic autoimmune inflammatory response following SCI that may contribute to further cell damage. In addition to autoimmunity, lymphocytes’ release of pro-inflammatory cytokines such as tumor necrosis factor-α (TNF-α), IL-2, and interferon gamma contributes to OL death [45]. Finally, matrix metalloproteinases produced by glia and inflammatory cells may contribute to demyelination [46]. Thus, the inflammatory response after SCI is a double-edged sword: while necessary for initial tissue clearance, it is a major impediment to OL and OPC survival and function, ultimately limiting remyelination and recovery.

### 2.2. Progressive Myelin Loss

Some studies suggest that demyelination continues chronically beyond secondary injury [47,48]. One study by Totoiu and Keirstead showed that following SCI in rats, demyelinated axons peaked at 1 day post-contusion, transiently declined by 7–14 days, then steadily rose through 450 days, whereas OL- and Schwann-cell-remyelinated axons appeared by day 14 but never fully compensated for ongoing myelin loss [48]. This pattern demonstrates that spinal cord contusion induces an acute demyelination phase followed by chronic, progressive demyelination despite partial remyelination, indicating that myelin breakdown is a prolonged pathological process. The findings conclude that effective therapies for spinal cord injury must directly address chronic demyelination to preserve white matter integrity and improve functional recovery [48].

Immunohistochemical analysis of seven chronically injured human spinal cords (1–22 years post-injury) showed that segmental demyelination of spared axons persists in most cases, especially at 1–2 years after injury. Adjacent to these bare axons, researchers found both typical bundles of peripheral myelin and more diffuse P0-positive myelin sheaths directly on central axons, showing that Schwann cells had migrated in and formed new myelin. These findings demonstrate that chronic demyelination contributes to long-term axonal dysfunction in human SCI, yet endogenous Schwann cells can invade astrocyte-poor niches and restore myelin, supporting Schwann cell–based strategies to enhance remyelination in chronic spinal cord injury [47]. While conduction through demyelination regions is possible, demyelinated axons have decreased conduction velocity and prolonged refractory periods and are more prone to physical and chemical damage [49]. Thus, persistent demyelination contributes to neurological and functional impairments following SCI.

## 3. Molecular Pathways Governing Myelination and Remyelination

**Figure 1:**
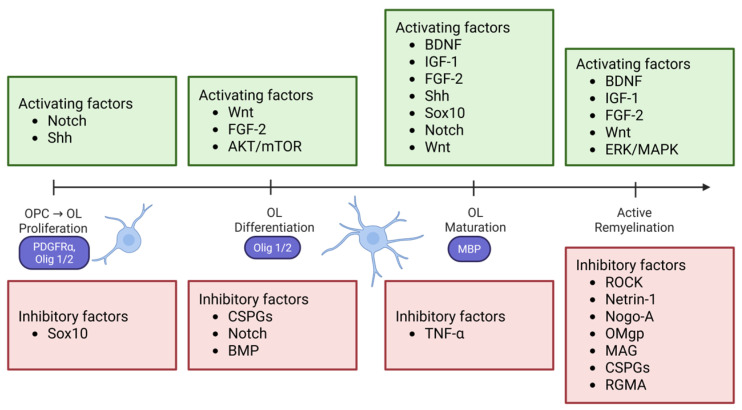
CNS remyelination with stage-relevant molecular messengers. Molecular markers specific to OL maturation are shown in the rounded purple text bubbles. Created in BioRender. Ahuja, C. (2025) https://BioRender.com/fz2chtn (accessed on 8 June 2025).

To appreciate the current approaches of remyelination, it is essential to first understand the molecular bases that govern myelination and remyelination. The pertinent molecular pathways that regulate these processes provide insight into mechanisms that can effectively modulate them. Here we discuss several key signaling pathways (Figure 1), transcriptional regulation, the role of microglia and astrocytes, lipid metabolism, and the crosstalk between some of these components.

### 3.1. Role of Microglia in Inflammation and Myelin Debris Clearing

Other glial cells, particularly microglia and astrocytes, also have important functions in myelination. Microglia are the resident macrophages of the CNS, existing in demyelinating pro-inflammatory microglia and reparative anti-inflammatory microglia [50].

Pro-inflammatory microglia are rapidly activated after SCI and play a dual role in remyelination. Initially, these cells are essential for the phagocytic clearance of myelin debris, which creates a favorable environment for OPC recruitment and subsequent differentiation [51]. A key experiment utilized a toxin-based spinal cord demyelination model in zebrafish and mice to demonstrate that these processes are governed by NF-κB- and MyD88-dependent pro-inflammatory activation, with decreased debris clearance and OL generation in MyD88-deficient mice [51]. Additionally, pro-inflammatory microglia secrete TNF-α, which can stimulate the generation of new premyelinating OLs [52]. However, with in vitro fetal CNS cells, excessive or prolonged pro-inflammatory signaling was cytotoxic to mature OLs and OPCs were especially vulnerable, ultimately hindering remyelination if not properly regulated [43,53].

Conversely, anti-inflammatory macrophages play a regenerative role in the myelination pathway. Miron et al. used in vivo depletion of M2-polarized cells in a mouse model of CNS demyelination and found that loss of these cells impaired OL differentiation and remyelination. In vitro, conditioned media from M2 macrophages/microglia enhanced OPC differentiation, and blocking M2-derived activin-A inhibited this effect [54]. While pro-inflammatory macrophages primarily establish the remyelination environment, anti-inflammatory macrophages secrete growth factors such as activin-A to directly promote OPC maturation and myelin repair. Additional studies conducted in vitro and in mice models have shown that the transition from pro-inflammatory to anti-inflammatory phenotypes is essential for optimal remyelination, as anti-inflammatory macrophages secrete trophic factors and matrix-remodeling enzymes that support OPC survival, proliferation, and differentiation [50,54,55,56,57].

### 3.2. OPC Migration

OPC migration is the initial step in the endogenous remyelination process following SCI. This migration of OPCs is tightly regulated by a network of signaling pathways that integrate both permissive and inhibitory cues from the injury microenvironment [22]. A major group of signaling pathways include the growth factors. For instance, PDGF-AA, acting via PDGFRα, is a principal chemoattractant for OPCs, promoting their directed migration toward demyelinated regions. Fibroblast Growth Factor (FGF) 2 also enhances OPC motility and is upregulated in the injured spinal cord, supporting recruitment to the lesion [58]. Neuregulin-1 signals through ErbB receptors and is essential for the transformation of OPCs into myelinating Schwann cells, a process that contributes to remyelination and functional recovery after SCI [59]. Disruption of ErbB signaling in PDGFRα+ OPCs with contusion-SCI mice models impairs both Schwann cell-mediated remyelination and locomotor recovery, highlighting the importance of this pathway in OPC plasticity and migration [60].

Downstream of the growth-factor receptors are the phosphoinositide 3-kinase and protein kinase B (PI3K/AKT) and Ras/MAPK cascades, which are essential for cytoskeletal remodeling and cell motility [61]. PI3K/AKT signaling promotes actin polymerization and cell survival, facilitating OPC migration in response to injury-induced cues. The Ras/MAPK pathway similarly regulates cytoskeletal dynamics and is required for efficient OPC movement toward demyelinated axons. Crosstalk between these pathways allows for fine-tuned responses to the complex milieu of the injured spinal cord [62].

Notch signaling, particularly through ligand-specific activation (e.g., Jagged1 and F3/contactin), has a context-dependent role in OPC migration. In the acute phase following injury, reactive astrocytes upregulate the Notch ligand Jagged1. This activation in OPCs both promotes their proliferation and maintains them in an undifferentiated, migratory state, thereby expanding the pool of OPCs available for recruitment to demyelinated regions. This effect is mediated by canonical Notch signaling, leading to upregulation of downstream effectors such as Hes5, which suppresses differentiation and supports continued migration and expansion of the OPC population in the lesion environment [63,64].

While there are numerous mechanisms promoting OPC migration to the injury site, the post-injury environment is also shaped by inhibitory molecules such as chondroitin sulfate proteoglycans (CSPGs) and pro-inflammatory cytokines, which are upregulated by reactive astrocytes and microglia. These activate intracellular inhibitors of migration, including RhoA, and restrict OPC entry into the lesion core [61]. Rho family GTPases (RhoA, Rac1, Cdc42) are central regulators of OPC polarity, migration speed, and directionality. These pathways integrate signals from the extracellular matrix and modulate actin cytoskeleton reorganization, which is crucial for OPC movement [61]. Inhibitory cues in the local microenvironment such as CSPGs, can overactivate RhoA and downstream effectors, thereby excessively increasing actomyosin contractility, thus impeding extension and restricting OPC entry into the lesion core [21]. The balance between these inhibitory cues and growth factor-mediated pro-migratory signals ultimately determines the extent and pattern of OPC recruitment after SCI.

Although CSPGs inhibit migration, RhoA signaling, mediated by RARα and RARβ, promotes OPC migration and is involved in neuron–glia crosstalk. Exosome-associated RA release and decorin-mediated pathways regulate OPC behavior in the injured spinal cord, influencing their ability to reach demyelinated areas. Neuronal RARβ activation induces decorin expression, which in turn regulates RA availability and RARα activation in OPCs, promoting their migration and maturation. Decorin also scavenges CSPGs and suppresses their synthesis, further removing barriers to OPC migration [65].

### 3.3. OPC Proliferation

Notch signaling is another pathway influencing myelination. Notch activation particularly promotes OPC proliferation while inhibiting OPC differentiation [63,66]. Thus, OPCs are maintained in a proliferative, undifferentiated state by the Notch pathway. In vivo studies inhibiting Notch1 signaling in OPCs have found significant increases in premature OL differentiation and ectopic OL placement in the SC gray matter, implying the role of Notch in spatial and temporal regulation of OL development [67]. Furthermore, astrocyte-derived Endothelin-1 has been shown to promote Jagged1 expression to bind a Notch receptor to then activate the Notch pathway, subsequently inhibiting remyelination [68].

Notch continues to play an important role in mature OLs by promoting maturation and subsequent upregulation of myelin-related proteins and myelin production. Alternate context-dependent pathways involving Deltex1 and F3/contactin have demonstrated variable effects of Notch depending on the ligand interactions, notably that Notch signaling is capable of promoting, rather than stalling, OL maturation [69,70,71].

The Sonic Hedgehog (Shh) pathway plays a vital role in SC dorsal–ventral axis formation and myelination in early and late development, respectively [72]. Using the Gli1 transcription factor, which regulates OLs maturation and myelin production, Shh promotes the proliferation and differentiation of OPCs to mature OLs in myelination [73,74]. In the context of remyelination, such as after SCI, Shh facilitates the repair of damaged myelin through its upregulation in OL lineage cells, promoting OPC proliferation to restore the myelin sheath and improve functional recovery [74,75,76]. Furthermore, Shh/Gli1 signaling is induced in reactive astrocytes after SCI, playing a crucial role in maintaining blood–SC barrier permeability [75].

Other pathways are mediated by growth factors, including Brain-Derived Neurotrophic Factor (BDNF), Insulin-Like Growth Factor-1 (IGF-1), and FGF. BDNF enhances OLs’ survival and increases the expression of MBP, a structural protein of the myelin sheath, to promote myelin sheath formation, axonal remyelination, and functional recovery after an SCI [76,77]. IGF-1, on the other hand, is intricately involved in the remyelination process, upregulated at peak demyelination in microglia and reactive astrocytes to promote myelin sheath formation and repair [78]. Finally, FGF, particularly FGF-2, is upregulated to promote balanced OPC proliferation and differentiation to repair damaged myelin sheaths in the remyelination process [78,79].

### 3.4. OPC Differentiation

The transition from OPC to mature, myelinating OL is governed by a complex interplay of intrinsic and extrinsic signals. An important route in OPC differentiation involves G protein-coupled receptors (GPCRs) and the downstream cyclic adenosine monophosphate (cAMP) signaling axis. GPCR activation can elevate intracellular cAMP, which in turn activates two major effectors: protein kinase A (PKA) and exchange protein directly activated by cAMP (EPAC). PKA phosphorylates a range of substrates, including the cAMP response element-binding protein (CREB), a transcription factor that promotes the expression of genes required for OL maturation and myelin gene transcription. EPAC, acting independently of PKA, also modulates cytoskeletal dynamics and gene expression, further supporting OPC differentiation. The balance of cAMP signaling is tightly controlled by phosphodiesterases (PDEs), which degrade cAMP and thus serve as regulators of the timing and extent of OPC maturation [80,81,82].

Evidence from MS and other CNS demyelinating models demonstrates that pharmacological modulation of cAMP/PKA/CREB signaling can enhance OPC differentiation and remyelination, suggesting translational potential for SCI. For example, PDE inhibitors have been shown to increase cAMP levels, promote CREB activation, and accelerate OL maturation in preclinical models. These findings underscore the relevance of cAMP pathway modulation not only in SCI but also in broader contexts of CNS repair [82].

The Wnt/ß-catenin pathway has multifaceted roles in myelination and remyelination. In early neural development, it is essential for the differentiation and maturation of OPCs, as previous work has correlated myelination delays with pathway disruptions during this time [83,84]. This pathway activates during response to SCI, but its overactivation after the acute phase has conversely been observed to inhibit OPC maturation and subsequently obstruct remyelination [83,85,86,87]. Consequently, further understanding of the temporal effects of this pathway is needed to understand potential therapeutic targets.

In general, the Wnt/ß-catenin pathway plays an essential role in both embryonic development and adult homeostasis. Pathway activation occurs when Wnt ligands bind to the Frizzled receptor and the LRP5/6 co-receptors. In the absence of activation, the ß-catenin destruction complex, consisting of Axin, APC, GSK3, and CK1, phosphorylates ß-catenin for ubiquitin-mediated degradation. Activation inhibits the ß-catenin destruction complex through the activation of Dishevelled, allowing the accumulation of ß-catenin. The ß-catenin can then enter the nucleus and interact with TCF/LEF and other transcriptions factors to promote the transcription of the target cell proliferation, specification, and differentiation genes [88,89,90].

The AKT/mTOR pathway is a central regulator of OPC differentiation and myelination in the CNS. Activation of the PI3K/AKT axis leads to downstream engagement of mTOR, which exists in two functionally distinct complexes: mTORC1 and mTORC2 [81]. mTORC1 is the primary driver of OL differentiation, promoting the transcription and translation of myelin genes, lipid biosynthesis, and the morphological maturation of OLs. This pathway integrates extrinsic signals such as growth factors (e.g., IGF-1, pleiotrophin, laminin-2) and intracellular cues to coordinate the transition from OPC to myelinating OL [91]. mTORC1 activity is required for the cap-dependent translation of myelin proteins and for the expansion of myelin sheaths, with both loss- and gain-of-function studies demonstrating that a balanced level of mTORC1 activity is essential for accurate myelination [92]. mTORC2, while less critical in the spinal cord, contributes to myelination in a region-specific manner, particularly in the brain [93].

Importantly, the AKT/mTOR pathway also interacts with other signaling cascades, such as ERK1/2-MAPK, to fine-tune the timing and extent of OPC differentiation and myelin sheath growth. Disruption of this pathway, either by genetic deletion or pharmacological inhibition (e.g., rapamycin), impairs OL maturation and myelin protein expression, while the activation of upstream effectors (e.g., via PTN-PTPRZ or Fyn signaling) enhances differentiation and remyelination [94].

In contrast, the bone morphogenetic protein (BMP) signaling pathway acts as a potent extrinsic inhibitor of OPC differentiation, particularly in the context of spinal cord injury. The BMP signaling cascade leads to the induction of inhibitors of differentiation (Id) proteins such as Id2. This diverts OPC fate away from OL lineage progression and toward astrocytic differentiation, thereby limiting remyelination potential [95]. The inhibitory effect of BMPs on OPC differentiation is particularly pronounced post-injury, where elevated BMP signaling contributes to the failure of endogenous and transplanted OPCs to generate mature OLs. Notably, there is significant crosstalk between the mTOR and BMP pathways: mTOR activity suppresses BMP signaling by upregulating FKBP12, a negative regulator of BMP receptor activity, and by altering the transcriptional complex at the Id2 promoter. Inhibition of mTOR leads to increased BMP/SMAD activity and Id2 expression, further blocking OL differentiation. Conversely, pharmacological or genetic inhibition of BMP signaling (e.g., with noggin) can rescue OL differentiation and remyelination in the injured SC [96]. Thus, the balance between AKT/mTOR-driven promotion and BMP-mediated inhibition of OPC differentiation is a critical determinant of remyelination efficiency after CNS injury.

### 3.5. Transcriptional Regulation

In the aftermath of SCI, injury-induced dysregulation of transcription factors and similar mechanisms often inhibits or prolongs the remyelination process. For example, OL-specific factors can heavily influence the maturation of OPCs and the expression of myelin genes. Olig1 and Olig2 are two specific factors that are critical for the specification and differentiation of OPCs. Olig2 has particularly been observed in this capacity with previous work showing that its overexpression has been correlated with enhanced OPC differentiation and remyelination, whereas its ablation has led to the inhibition of these same processes in immature OPCs [97,98]. Meanwhile, Olig1 is especially important for OPC differentiation and myelination with previous work showing its required expression for the repair of demyelinated CNS lesions and its overexpression promoting further differentiation and remyelination [99]. When Olig1 and Olig2 are simultaneously expressed, significant increases in OPC proliferation and maturation have been seen [100].

Sox10 is another OL-specific transcription factor that has several key roles influencing OPCs and myelination. It regulates myelin-related genes, working with other transcription factors like Olig1 to transcribe MBP [101]. To further maintain the myelination process, Sox10 enables the survival of myelinating OLs after they have wrapped axons [102]. Moreover, Sox10 is essential for OLs identity and can promote the generation of new OLs from different non-OL lineages such as in its ability to reprogram astrocytes [103,104].

Nkx2.2 is a transcription factor upregulated in OPCs and OLs around demyelination and has been shown to coincide with OPC differentiation [105,106]. Moreover, previous work has shown the interaction of Nkx2.2 with Olig2 to enhance myelin gene expression, promoting the formation of myelin sheaths in the remyelination process [50,107]. While inflammatory cytokines and conditions can hinder Nkx2.2 expression, microenvironmental regulation strategies can be used to mitigate these effects [21,108].

ID2 and ID4, members of the inhibitor of DNA binding (ID) family, act as negative regulators of OPC differentiation. ID2 inhibits oligodendrogenesis by interacting with OLIG proteins and the retinoblastoma tumor suppressor, with its expression modulated by Wnt signaling and histone deacetylase (HDAC) activity [109]. Although ID4 functions similarly, genetic studies suggest that it is essential for oligodendrogenesis, whereas ID2 is largely dispensable. Notably, ID4 deletion alone does not impair OL differentiation, and compound ID2/ID4 knockouts produce only mild, transient effects [110].

While forced overexpression of either ID2 or ID4 potently suppresses OPC differentiation in vitro, in vivo studies indicate they are not the primary repressors of this process during development. However, ID2 overexpression in neural stem cells can arrest OPC maturation and contribute to tumorigenesis within PDGF-rich environments, underscoring its potential pathological significance. For normal OPC differentiation to proceed, downregulation of ID2—and to a lesser extent, ID4—is required, a process tightly regulated by HDAC activity [111].

Tcf4 is a class I basic helix–loop–helix (bHLH) transcription factor that acts as the preferred heterodimerization partner for Olig2 in OLs [112]. This heterodimerization is essential for the activation of myelin gene enhancers and is required for proper terminal differentiation and myelination of OPCs. Genetic ablation of Tcf4 in OL lineage cells leads to impaired differentiation and myelination, with only partial compensation by its paralog Tcf3. Mechanistically, Tcf4–Olig2 complexes directly regulate the transcription of key myelin genes, and this interaction assists with controlling OL maturation [113].

Sox11 is a member of the SoxC family and functions as a negative regulator of OPC differentiation. High Sox11 expression is characteristic of early OPCs, and its downregulation is critical for the transition to mature, myelinating OLs. Persistent Sox11 expression inhibits differentiation, while its timely repression allows for the activation of myelin gene expression and progression through the lineage [114]. Sox11 thus acts as a developmental brake that must be released for effective myelination.

Hes5 is a bHLH transcriptional repressor and a canonical Notch pathway effector. In OPCs, Hes5 participates in cross-regulatory interactions with Olig2 and Sox10, forming part of a network that balances proliferation and differentiation. Hes5 represses myelin gene expression and antagonizes the activity of Olig2 and Sox10, thereby maintaining OPCs in an undifferentiated state. Downregulation of Hes5 is necessary for the initiation of differentiation, and its persistent expression can block myelination [115]. These cross-regulatory interactions ensure the precise timing of OPC maturation and myelin gene activation.

### 3.6. Role of Astrocytes in Myelination

Like microglia, astrocytes can have positive and negative influences on myelination. OPC differentiation is promoted through secretion of FGF-2 by astrocytes and the upregulation of cholesterol synthesis and export from astrocytes supports myelin fabrication [116]. Recent evidence in focally-lesioned mice models demonstrates that the remyelinating astrocyte–OL interactions are essential for the survival and maturation of regenerating OLs. Such interactions occur via downregulation of the nuclear erythroid 2-related factor (Nrf2) pathway, which increases astrocytic cholesterol biosynthesis and efflux, and disruption of this pathway impairs remyelination, while its stimulation restores myelin repair [117]. The role of astrocytic cholesterol has been characterized in early developmental myelination, but its role in remyelination is unclear [117,118].

Conversely, reactive astrocytes can inhibit remyelination by releasing molecules in mice contusion models such as bone morphogenetic proteins (BMPs) and CSPGs, which suppress OPC differentiation and promote astrocytic rather than OL lineage commitment [21,95]. Astrocyte-derived extracellular vesicles (EVs) also modulate the microenvironment, with non-reactive astrocyte vesicles supporting OPC maturation and functional recovery, while vesicles from reactive astrocytes exacerbate inflammation and impede remyelination [119]. Thus, the net effect of astrocytes on remyelination after SCI is determined by their activation state and the balance of the supportive versus inhibitory signals they provide to OPCs and OLs.

### 3.7. Role of Lipid Metabolism and Turnover in Myelination

Lipid metabolism and turnover in both immune cells and OPCs are essential for effective remyelination after SCI, when macrophages phagocytose myelin debris, which is rich in cholesterol and other lipids. To adapt to this lipid load, these phagocytes upregulate cholesterol esterification and lipid droplet biogenesis, a process that is critically dependent on TREM2 signaling. Failure, as seen in TREM2-deficient mouse models, leads to unresolved endoplasmic reticulum stress, impaired debris clearance, and ultimately failed remyelination. Subsequent pharmacological alleviation of ER stress restored lipid droplet formation and the regenerative response, demonstrating the necessity of lipid metabolism in immune cells for myelin repair [120]. Furthermore, chronic SCI in a hemicontusion mouse model led to progressive lipid accumulation in myeloid cells, which contributed to additional inflammation and impaired remyelination [121].

In OPCs, lipid metabolism is equally important. The transition from OPC to mature OL requires extensive upregulation of lipid biosynthesis, as myelin membranes are highly enriched in cholesterol and specific phospholipids. In vivo studies using the chronic cuprizone demyelination model in mice have shown that OPCs and OL lineage cells upregulate cholesterol biosynthesis gene pathways during the remyelination phase [122]. While overall cholesterol synthesis is required for myelin membrane formation in vivo, in vitro studies suggest that remyelination and OPC differentiation can be promoted by blocking specific enzymes in the cholesterol biosynthesis pathway, leading to the accumulation of 8,9-unsaturated sterol intermediates. Buildup of sterols then stimulates OL formation from OPCs, and this is validated by in vivo mice demyelination models [123].

## 4. Challenges in Remyelination

### 4.1. Inhibitory Microenvironment

Beyond the damage and demyelination that occurs during primary and secondary injury, challenges to remyelination further complicate recovery after SCI. Myelin-associated inhibitors (MAIs) including Nogo-A, myelin-associated glycoprotein, and OL myelin glycoprotein are released by OLs following SCI and create a microenvironment inhibitory of remyelination [38]. These three classic MAIs inhibit the anatomical rearrangements and plasticity necessary for regrowth and remyelination [124]. Netrin-1 has also been identified as a novel MAI, and further studies characterizing other MAIs and their specific functions will further the understanding of the inhibitory microenvironment post-SCI [125].

OPCs play the primary role in remyelination, and many mechanisms of remyelination inhibition act on OPCs [20,126]. Astrocytes undergo phenotypic changes in response to injury during a process called reactive astrogliosis, becoming reactive astrocytes and then scar-forming astrocytes that contribute to the glial scar, a protective response to CNS damage [127]. Reactive astrocytes release bone morphogenetic proteins (BMPs), which inhibit OPC differentiation, and TNF-α, which induces OPC apoptosis and prevents OPC maturation [95,128]. Glial scars physically block axonal regrowth and release inhibitory factors such as CSPGs [34]. Research has consistently demonstrated the inhibitory role of CSPGs in axonal regeneration, conduction, and remyelination [34]. CSPGs inhibit OPC process outgrowth, differentiation, and migration, furthering the inhibition of remyelination after SCI [129,130].

### 4.2. Impaired Recruitment and Differentiation of OPCs

Given the crucial role of OPCs in remyelination, anything that inhibits OPC migration or differentiation will impair remyelination. Reactive astrocytes stimulated by chronic inflammatory signals play a key role in interfering with OPC differentiation through the release of TNF-α [128]. These reactive astrocytes also upregulate the expression of BMP, which promotes OPC differentiation to astrocytes rather than OLs, thus reducing the number of OLs that can contribute to remyelination [95]. Reactive astrocytes can also directly induce apoptosis of OPCs, further interfering with remyelination [128].

The signaling pathways involved in response to SCI are complex and interconnected. However, a few pathways have been identified as particularly relevant to OPC function following SCI. The Notch1 pathway is permissive of OPC proliferation, but inhibitory of OPC maturation, thus inhibiting remyelination [64]. Other pathways involved in OPC differentiation and function are the Wnt/β-catenin pathway, PI3K/AKT/mTOR pathway, and ERK/MAPK pathway [62]. The Wnt/β-catenin and AKT/mTOR pathways play direct roles in OPC differentiation and myelination, and the ERK/MAPK pathway regulates myelin sheath expansion [62]. Dysregulation of these pathways can impair remyelination, so these pathways serve as potential therapeutic targets for SCI.

### 4.3. Chronic Inflammation

Acute inflammation has been shown to stimulate remyelination by stimulating OPCs [131]. Researchers used the taiep rat model, a myelin-deficient rodent that exhibits chronic demyelination without acute inflammation, combined with X-irradiation and transplantation of OPCs to demonstrate that acute inflammation stimulates remyelination in previously non-remyelinating, astrocytosed CNS tissue [132]. However, chronic inflammation can impede this process. In addition to activated astrocytes, infiltrating immune cells also contribute to the hostile microenvironment following SCI and can restrict the differentiation and activity of OPCs [21]. Persistence of myelin debris after injury results in further delayed recruitment of macrophages, contributing to chronic inflammation and inhibition of remyelination [133].

Following injury, macrophages can differentiate into the inflammatory or anti-inflammatory phenotype, thus promoting either an injury or repair process [134,135]. Inflammatory macrophages are associated with higher production of CSPGs and repulsive guidance molecule A (RGMA) compared to anti-inflammatory macrophages [34,136]. RGMA has been demonstrated with a T8 four-fifths’ overhemisection SCI rat model to induce axonal retraction following SCI [136]. Inflammatory macrophages predominate over anti-inflammatory macrophages in CNS injury, mediated by TNF-α and iron, two products present in abundance following SCI [45]. The predominance of inflammatory macrophages promotes an inflammatory state through production of CSPGs and repulsive factors, thus inhibiting remyelination [34].

### 4.4. Axonal Degeneration

Myelin plays an important role in maintaining the integrity of axons through both physical protection and metabolic support [137]. OLs provide glycolysis byproducts to axons, supply trophic factors, assist with potassium buffering, and protect neurons from oxidative stress [136]. With the supportive role of OLs and the myelin sheath, demyelination and impaired remyelination will hinder neuronal function and repair. In the absence of protective and supportive myelin, axons are more prone to damage, after which they undergo Wallerian degeneration distally and axonal retraction proximally [138]. Thus, impaired remyelination following SCI threatens neuronal stability and survival.

### 4.5. Practical Challenges in Functional Recovery

#### 4.5.1. Variability in Outcomes

Recovery following SCI varies depending on severity and level of injury. A meta-analysis examining outcomes following traumatic SCI found that more severe injuries consistently have poorer neurological recovery rates compared to less severe injuries, and injuries at the cervical level have poorer recovery than those at the lumbar level [139]. Results for the degree of functional recovery are similar, with patients sustaining incomplete initial injuries (ASIA Impairment Scale (AIS) score of B or C) having greater recovery than those with complete initial injuries (AIS score of A) [140]. Data also suggest that hemorrhage and rostral–caudal length of edema are important to outcomes following SCI. Patients without SC hemorrhage had better mobility recovery than those with hemorrhage, and a greater rostral–caudal spread of edema was associated with poorer functional recovery [141]. Additionally, locomotive central pattern generators (CPGs) play a key role in functional recovery, especially when preserved post-SCI. CPGs are intrinsic spinal circuits capable of generating rhythmic locomotor activity and may support recovery even when supraspinal input is lost. In both animal models and humans, CPGs can be activated by sensory feedback and neuromodulation, enabling basic stepping and locomotor patterns even after complete SCI [142,143]. Rehabilitation strategies that harness CPG activity, such as locomotor training and electrical stimulation, can facilitate recovery of walking function by promoting reorganization and plasticity within spinal circuits [144].

Due to the heterogeneous nature and severity spectrum of SCI, targeted therapies are the best way to achieve neurological and functional recovery. Most SCI treatment today is supportive and focuses on minimizing progression of injury and preventing further injury through spinal stabilization and avoidance of hypotension and hypoxia [145,146]. However, these treatments do not have regenerative effects. Newer therapeutic methods including cell transplant and targeted pharmacologic treatment may be the next steps towards developing regenerative SCI treatments [146]. Cell therapies involving transplant of one or more cell types to injured areas show potential for achieving regeneration following SCI [147]. Current cell types being investigated include OPCs, embryonic stem cells, induced pluripotent stem cells (iPSCs), neural progenitor cells (NPCs), and mesenchymal stromal/stem cells (MSCs) [147]. Transplanted OPCs have the potential to aid in remyelination, transplanted MSCs may provide trophic support, and MSCs and NPCs can aid in immune modulation [147]. The transplant of OPCs and NPCs has shown promise in promoting remyelination and repair in animal models, but more research needs to be conducted to translate such therapies to humans [148]. In addition to cell therapies, pharmacologic therapy may be able to aid in remyelination through the targeting of key molecules involved in pathways involved in OPC recruitment and differentiation [7].

#### 4.5.2. Recovery and Long-Term Stability

While promotion and acceleration of remyelination following SCI is a promising therapy, remyelination alone is not enough to promote full recovery to pre-injury function [147]. Future therapies aiming to restore pre-injury function will need to address a combination of factors contributing to demyelination, as well as prevention of remyelination [149]. A multifaceted approach aimed at reducing secondary injury mechanisms including inflammation, excitotoxicity, and oxidative stress would need to be coupled with regenerative therapies that promote the regeneration of both myelin and neurons to achieve the goal of restoring pre-injury functionality after SCI.

## 5. Therapeutic Strategies for Promoting Remyelination in SCI

Remyelination provides many potential benefits in the recovery of individuals with SCI. Enhanced myelination of corticospinal and motor pathways improves voluntary muscle control, coordination, and fine motor skills. These improvements facilitate functional recovery to enable further activities of daily living, such as writing or grasping objects. Remyelination of sensory pathways restores the transmission of proprioceptive, tactile, and pain signals. Recovered sensory function can improve both balance and spatial awareness, further enhancing motor recovery. Previous work has demonstrated numerous sensorimotor improvements associated with remyelination after SCI [149].

Sensorimotor recovery is essential, but restoration of autonomic functions, such as lower urinary tract, bowel, and sexual functions, is less often examined in research despite being ranked as a high priority in patients with SCI [150,151,152]. Increased research is needed into the potential of remyelination to restore neuronal function in these autonomic pathways. The following subsections will elaborate which candidate therapies have been explored in animal and human models, with further clinical trial data presented in Table 1.

### 5.1. Pharmaceutical Therapies

Various pharmaceutical strategies can be utilized to modulate myelination. Therapies that target myelin inhibitors are one such example, including anti-Nogo-A antibodies and Rho-Associated Kinase (ROCK) inhibitors. Anti-Nogo-A antibodies block Nogo-A, a myelin-associated inhibitor protein which inhibits neuronal growth and plasticity [158]. Consequently, axonal sprouting, regeneration, and remyelination are promoted. In fact, studies have shown that anti-Nogo-A therapies increase axonal sprouting and arborization, especially in the corticospinal tract, while also increasing remyelination without affecting OPC proliferation or differentiation [158]. Animal models have suggested that anti-Nogo-A therapies reduce neuroinflammation and increase neuronal survival, but the potential efficacy may be temporally and spatially dependent relative to the injury site [159,160]. While clinical trials have demonstrated their safety in treating SCI in humans, further research is needed to delineate the efficacy of these drugs [154,155].

On the other hand, ROCK inhibitors inhibit the Rho/ROCK pathway, which normally increases neuronal apoptosis, neuroinflammation, and axonal growth inhibition after SCI. Through modulation of cytoskeletal dynamics and reduction in growth cone collapse, ROCK inhibitors promote axonal regeneration, neurite growth, and remyelination [161,162]. These alterations enhance OPC migration and differentiation to promote remyelination. Preclinical animal trials and preliminary clinical trials have shown promise, but more work is needed to optimize treatment protocols and reduce side effects [162,163,164,165].

There is therapeutic potential in modulating microglial activation states using cytokines and pharmaceuticals. Minocycline, a tetracycline derivative, creates an anti-inflammatory environment that promotes remyelination by inhibiting microglial activation, reducing pro-inflammatory cytokines, inhibiting apoptosis, and reducing oxidative stress [156,166,167,168]. Similarly, previous work has shown that TGF-ß, IL-4, IL-10, and IL-13 shift microglia to an anti-inflammatory phenotype, leading to reduced inflammation, enhanced differentiation, and increased repair and remyelination [57,169,170,171,172]. The combination of LPS, IL-4, and IL-13 has particularly been shown to show these remyelination enhancing effects in an animal model [57]. Beyond cytokines, pharmaceutical approaches that can activate microglia and promote the regenerative phenotype to enhance myelination include P2Y12 antagonists, Amphotericin B combined with MCSF, and more [157,173,174].

Epigenetic modulators, such as HDAC inhibitors, alter the acetylation status of histones to influence chromatin and transcription states. Specifically, HDACs promote remyelination by increasing the expression of myelin-associated genes in OPCs, facilitating their differentiation into mature OLs. Preclinical studies have demonstrated the abilities of HDAC inhibitors, such as valproic acid and CI-994, in reducing inflammation, enhancing remyelination, and improving outcomes [175,176,177]. In a similar fashion, DNA methyltransferase (DNMT) inhibitors prevent the epigenetic silence of key remyelination genes, enabling increased OPC maturation, enhanced myelin regeneration, and improved functional outcomes as seen in preclinical studies [178,179]. While DNMT inhibitors have shown promise in various neurological conditions, more research is needed to establish their safety and efficacy in treating SCI in humans [180].

Consequently, pharmaceuticals in the form of Shh agonists have been shown to promote remyelination and functional recovery after SCI [181,182]. Shh agonists primarily targeted Smoothened within Shh to lead to downstream activation of Gli1 [73,182,183]. While the complete modulatory effects of the Notch pathway on remyelination are uncertain, Notch inhibition through gamma-secretase inhibitors has shown promise in enhancing remyelination [64,68].

Several therapeutic remyelination approaches targeting the Wnt/ß-catenin pathway involve pharmacological inhibitors and small interfering RNAs (siRNAs) to attenuate excessive Wnt/ß-catenin signaling. Some inhibitors include XAV939, which functions by stabilizing Axin2 to promote ß-catenin degradation, and inhibitors of the Daam2-PIP5K pathway, a promoter of Wnt signaling [85,184]. Approaches using siRNAs can target molecules like ß-catenin or Celsr2, while other methods include employing pharmaceuticals, such as Indomethacin, or targeting key pathway molecules, such as Frizzled 1 and Wnt1 [86,185,186,187].

Another subdivision of these therapies are neuroprotective agents, including antioxidants and sodium channel blockers. Antioxidants, such as edaravone, mitigate oxidative damage to OLs and myelin. As free radical scavengers, antioxidants neutralize ROS to reduce lipid peroxidation and oxidative damage, creating an environment that is more favorable for the differentiation and maturation of OPCs, thereby promoting remyelination [188,189]. On the other hand, sodium channel blockers reduce excitotoxicity, creating an environment more conducive to OPC differentiation and subsequent remyelination, though substantial preclinical work is still needed to prove viability in humans [190,191,192].

The final subgroup discussed in this review are pharmaceuticals that facilitate growth factor delivery. Hydrogels and nanoparticles are often used for localized delivery of these growth factors, minimizing systemic side effects and maintaining therapeutic concentrations at the injury site [193,194,195]. Improved functional outcomes and increased myelination have been observed with several growth factors, including IGF-1, BDNF, and FGF, though there is a limited amount of human trials [196,197,198,199,200].

### 5.2. Non-Pharmaceutical Therapies

Beyond pharmaceutical methods, there are several non-pharmaceutical therapies that can be pursued for remyelination. Stem cell-based therapies, such as with OPC transplantation or iPSCs, are one subcategory of these non-pharmaceutical therapies. OPCs are transplanted to replenish the pool of myelinating cells. Preclinical studies show improved remyelination and functionary recovery with OPC transplantations. Still, cell survival, integration, and immune rejection remain as challenges [201,202,203,204]. However, the risk of immune rejection is reduced with iPSCs due to the autologous source of cells. These iPSCs can differentiate into OPCs or neural stem cells with previous animal model research showing functional recovery and remyelination through both routes [205,206].

Gene therapies are another non-pharmaceutical therapy modality. In these approaches, viral vectors are utilized to deliver genes encoding growth factors, anti-inflammatory cytokines, or transcription factors to injury sites to promote remyelination. For example, CRISPR/Cas9 technology has been used to edit human embryonic stem cell-derived OPCs to increase their remyelination abilities by reducing their sensitivity to a lesion chemorepellent [207]. Alternatively, RNA interference-based strategies can be employed as Sun et al. shows in their inhibition of Nogo with short hairpin RNA, similarly improving remyelination [16].

### 5.3. Emerging Therapies

While there are numerous established remyelination approaches, advances in biomaterials and tissue engineering have expanded methodologies to promote myelination, particularly with scaffolds and hydrogels. Scaffolds provide structural support and guidance for the regeneration of axons, successfully improving remyelination and functional outcomes after SCI in several cases [208,209,210]. Meanwhile, hydrogels enable the controlled delivery of growth factors or cells to provide similar benefits for remyelination [193].

Another emerging advancement is EVs derived from mesenchymal stem cells or OPCs and that can deliver cargo, such as RNA or proteins, to injury sites to promote remyelination [211,212,213]. The scalability and non-immunogenic nature of EVs are especially advantageous and promising for clinical use [211]. Yet another sector being explored for remyelination is nanotechnology. Nanoparticles allow targeted drug delivery that minimizes off-target effects and maximizes therapeutic concentrations at the injury site. The variety of nanoparticles is extensive, ranging from nanoparticles for growth factors to magnetic nanoparticles that can guide cells or drugs to specific regions with external magnetic fields [194,195,214,215]. The last emerging therapy category discussed in this review includes gene editing and epigenetic regulation. Some gene editing therapies utilize CRISPR/Cas9 technology as was the case in Wagstaff et al. with the editing of OPCs to artificially reduce an inhibitory environment [207]. With epigenetic regulation, one notable study identified a small-molecule epigenetic-silencing-inhibitor that induces an active chromatin landscape to activate myelin-associated pathways to enhance remyelination [209].

### 5.4. Therapeutic Markers of Remyelination

Non-invasive physiological measurements, such as visual evoked potentials, have been validated as quantitative biomarkers in both preclinical and clinical settings [216,217]. More invasive approaches, such as detecting cytokines including IL-6, IL-8, and IL-10 in blood or extracellular fluid, have also been employed in both animal models and human subjects; however, these markers lack specificity for SCI and tend to reflect a broader systemic inflammatory state [218]. Emerging methods, such as the analysis of microRNAs, offer promising advantages due to their stability and tissue-specific expression patterns, potentially providing a more accurate picture of SCI progression and recovery [219]. Molecular markers like macrophage GIT1 have also been explored, but many such candidates remain difficult to measure in practice and lack robust validation [220].

### 5.5. Translational Barriers in Remyelination Therapy

Though there are validated animal model preclinical studies of remyelination therapy, a major hurdle in translational research is the trustworthiness of preclinical models into the human clinical realm, as seen in Table 2. Animal models do not often replicate the complexity and heterogeneity of human SCI due to differences in immune response which ultimately impact the microenvironment surrounding the lesion [221]. Specifically, cell-based therapies which have proven effective in preclinical models have led to concerns of tumorigenicity and immune rejection in human SCI [222]. Furthermore, the methods used to assess endogenous remyelination and clinical outcomes do not necessarily translate to humans in a meaningful manner, complicating the therapeutic evaluation process [216,223].

## 6. Future Directions

### 6.1. Alternative Pathways

As discussed, the development of SCI occurs in two distinct phases. The PI3K/AKT pathway is linked with multiple underlying processes of secondary SCI, and, as such, is a possible area for treatment. PI3K is a lipid kinase that plays an important role in modulating cell proliferation, differentiation, and apoptosis via its own activation of AKT [224]. With respect to SCI, the PI3K/AKT plays a different role in the phases of SCI; it primarily plays a role in the subacute and chronic stages. In the subacute phase, it has been observed that PI3K/AKT activation can decrease inflammation and apoptosis by inhibiting toll-like receptor 4. This inhibition helps to alter macrophage phenotype to the more pro-inflammatory type and activates nuclear factor kappa-B (NF-κB), leading to subsequent pro-inflammatory compound release [224]. Indeed, PI3K/AKT activation itself can directly encourage microglia polarization towards the anti-inflammatory phenotype to further reduce inflammation. With respect to apoptosis, activation of this pathway can limit expression of pro-apoptotic enzymes like caspase-9 and caspase-3 [176]. Thus, in the subacute phase, activation of the PI3K/AKT pathway can counteract the apoptosis and inflammation specific to this phase of SCI. However, as PI3K/AKT activation drives scar formation in the chronic phase, the inhibition of this pathway during this period can help to limit such formation, promoting better axonal recovery.

While the PI3K/AKT pathway is certainly an intriguing area of research and poses the possibility of it playing a role in SCI treatment, currently there are certain limitations to the research that has been conducted on it. A review by Xiao et al., 2022, indicated that analysis of this pathway remains largely at a cellular phase, and more needs to be done at an animal and clinical level to truly elucidate the effectiveness of treatments targeting this pathway [225]. Additionally, this pathway has been studied in isolation, so whether it has any interaction with other SCI-related pathways is something that must be understood; moreover, the causality of whether PI3K/AKT activation plays a role in causing SCI or SCI causing PI3K/AKT activation should be clarified, as well.

Another possible pathway in SCI that can be targeted for treatment features Nrf2 and glutathione peroxidase 4 (GPX4). GPX4 plays an important role in apoptosis regulation and Nrf2 is a regulator of GPX4 [226]. It is thought that together this pathway plays a role in regulating ferroptosis, which is a kind of apoptosis associated with iron and lipid peroxidases. This cellular process has been shown to occur in SCI, contributing to the damage that occurs in the acute phase of the injury. Thus, curbing this process from occurring represents an alluring therapeutic target. An experiment using mice conducted by Ge et al. in 2021 [226], seemed to support this, finding that zinc can reduce ferroptosis and contribute to neuronal survival. They explain that GPX4 expression is blunted when SCI occurs, which leads to an increase in cell death. By using zinc, they were able to fix this decrease in activity, as the zinc was found to increase Nrf2 expression. Furthermore, exogenous zinc was able to decrease inflammation, as well as limit mitochondrial damage due to the reduction in oxidative product accumulation. While this is promising for potential therapy for SCI, as zinc is used in a slew of molecular processes, the exogenous addition of it may interfere with other pathways, so further research is needed.

Two other potential pathways of intervention are the NF-κB and p38-map kinase pathways. The former plays a role in augmenting microglial induced inflammation via transcription of pro-inflammatory cytokines, and the latter, when activated, can produce a large level of nitric oxide synthase that leads to reactive oxidative species production through nitric oxide [225]. The inhibition of the NF-κB and p38-map kinase pathways may alleviate cellular damage during initial SCI. In fact, an extract from Lamiophlomis rotate Kudo, forsythoside B, has been shown to do just this. Xiao et al. (2022) [225] showed that this extract has a neuroprotective effect in mice with SCI, as it downregulates both pathways, which limits apoptosis and neuroinflammation, decreasing overall neuronal damage.

### 6.2. Rehabilitation and Combinatorial Strategies

#### 6.2.1. Physical Rehabilitation

In addition to the host of therapeutic interventions that can promote remyelination, there are a number of other therapies that may also be beneficial in the recovery of patients with SCI. These include physical rehabilitation, which involves exercise and functional electrical stimulation, magnetic stimulation via an epidural or transcranial means, stem cell therapy, pharmacologic therapy, and then differing combinations of these therapies.

Exercise is a promising treatment for SCI due to its positive effects on the CNS, likely improving prognosis through activity-induced neurotrophic factor release. BDNF and IGF-1, which decline at the injury site, can be restored through exercise, particularly aerobic activity. Their recirculation is dose-dependent, influencing neuronal growth and survival. Increased IGF-1 levels activate the PI3K/AKT pathway, reducing endothelial apoptosis and promoting corticospinal tract axon regeneration [209].

Additionally, exercise promotes neuronal stem cell proliferation, aiding nervous system remodeling. Notably, neuronal precursor cells (NPrCs) differentiate into OLs, which remyelinate damaged cells post-SCI. Treadmill training expedites OL maturation, strengthens myelin, and increases myelin-related protein expression, enhancing sensory signal transmission in the SC [227]. Further studies confirmed this notion, and incidentally found that in addition to aiding in the remyelination effort after SCI, exercise pre-SCI may alter the SC environment to be more pro-myelination [228].

Exercise has been shown to directly enhance remyelination in animal models of myelinating disorders such as MS, and emerging evidence suggests similar benefits in humans. In murine models of demyelination, voluntary wheel running and aerobic exercise increase OPC differentiation, oligodendrogenesis, and the rate and extent of remyelination, with a role for OL PGC1α in mediating these effects [12,229,230]. Exercise also promotes the clearance of inhibitory lipid debris and upregulates pathways involved in oxidative stress response, metabolism, and synaptic transmission, all of which support a pro-remyelinating environment [231].

Functional electrical stimulation (FES) of the nervous system has been shown to have similar effects on OL proliferation post-SCI. A study examined the combined effects of human spinal neuronal progenitor cells (sNPCs) and tail electrical stimulation in rats. Results showed that this combination led to a greater differentiation of sNPCs into OLs than either treatment alone [232]. Additionally, rodents receiving both interventions had increased myelin compared to controls. Further, a 2023 review article found that FES promotes the maturation of NPrCs into OLs in the lumbar SC [233]. Interestingly, while there was an overall increase in OLs across the entire SC, they were not always primarily located at the site of injury. Therefore, both exercise and FES seem to aid in SCI recovery through their promotion of OL maturation.

With respect to the ultimate functional effects of these interventions on patients with SCI, a meta-analysis on the benefits of physical rehabilitation found that FES was primarily used to increase upper limb function; however, there was little effect in lower extremity independence and quality of life [234]. They found that walking on a treadmill with robotic assistance had a significant benefit on upper extremity independence compared to patients who simply walked on a treadmill though, surprisingly, both treadmill walking with and without robotic assistance showed no effect on walking capacity in SCI patients. This seems to conflict with the physiologic basis of exercise discussed above, but the researchers indicated this finding may have been a result of differing approaches to exercise. Nevertheless, this demonstrates the need for more investigation to determine how effective exercise can be as a treatment for patients with SCI.

#### 6.2.2. Magnetic Stimulation

The use of either epidurals or transcranial magnetic stimulation (TMS) represents yet another intervention. As previously discussed, SCI results in a significant amount of axonal damage to the corticospinal tract. Fortunately, there is evidence that demonstrates the use of TMS can reverse this process and lead to axonal growth and sprouting along this tract of nerves. A study found that SCI-induced rats treated with TMS had significantly better motor function, a more intact corticospinal tract, and axonal regeneration compared to controls. In addition, they observed that two axonal growth promoters (growth-associated protein 43 (Gap43) and synapsin I) and one axonal growth inhibitor (Nogo-positive cells) were increased and decreased, respectively, post-TMS [235]. Another rodent study observed that epidural oscillating fields had a significant positive effect on axonal preservation in the areas of white matter damage in addition to an increase in Gap43 [236].

TMS, particularly low-intensity repetitive TMS, has been shown to directly enhance remyelination in animal models of demyelinating disorders such as MS. This form of TMS delivered as intermittent theta burst stimulation to the cuprizone mouse model increases the number and length of myelin internodes produced by newly generated OLs and promotes the survival of mature OLs that support remyelination. This results in a greater proportion of axons being remyelinated in affected brain regions, such as the primary motor cortex and corpus callosum, following demyelination. The mechanism is thought to involve modulation of cortical neuronal activity, which in turn influences OPC differentiation and the behavior of both new and surviving OLs, paralleling the activity-dependent myelin plasticity observed with exercise interventions [237].

In human studies, TMS does seem to have positive effects in regard to walking speed and lower extremity function [234]. Additionally, one study showed that high-frequency TMS can increase the strength of lower limb muscles post-SCI and improve the plasticity of synapses [238,239]. Intriguingly, TMS seems to have a metabolic effect in SCI patients, as high-frequency TMS has been shown to modulate regional glucose metabolism, help insulin resistance, and moderate obesity [234]. TMS is an exciting potential intervention due to its non-invasiveness with the caveats of the therapy requires in-person appointments and more research is needed to fully understand the underlying effects and outcomes.

#### 6.2.3. Combinatorial Approaches

Beyond physical rehabilitation and magnetic stimulation, there remains the mechanistic field of regenerative medicine involving cell-based therapies and bioactive materials. While limited when used in isolation, they show promise in a combinatorial approach. Recent studies have investigated the role of neural stem cells, olfactory ensheathing cells, mesenchymal stem cells, and bone-marrow stem cells for this purpose. While stem cell therapies are a promising idea mechanistically, research on their effectiveness in humans remains limited; however, studies in animal models of SCI show functional improvements [240]. Several challenges hinder translation to clinical use, including the limited availability of stem cells and their low survival rate after implantation. As a result, not only are these cells difficult to obtain, but few successfully function once implanted.

Similar to stem cell therapy, the use of pharmacologic and biologic therapies shows some promise but are also limited in their effectiveness and practicality. Unlike stem cell therapy, there is more evidence supporting the role of these interventions in promoting functional recovery and quality of life [240]. However, a 2024 review of pharmacologic therapies concluded that previously conducted clinical trials have not shown clinically significant improvement in SCI patients despite some therapeutic promise in animal models [241]. A significant barrier in delivering medications, regardless of whether humans or animals are being studied, is the blood–spinal cord barrier. While the barrier can be broken down in the early phases of SCI, potentially allowing for effective drug delivery, the barrier begins to reform too quickly to promote the lengthy process of nervous tissue maturation and healing [240]. There remains the possibility of intrathecal injections, though cerebrospinal fluid flow could wash the agents away from their target site and limitations in drug half-life could make long-acting therapies challenging to apply.

Biomaterials, structurally strong synthetic or natural polymers, offer a potential scaffold for drug- or cell-based therapies [242]. Clinical trials have shown promise in using them to deliver cells for functional recovery in SCI patients, while non-clinical studies suggest their effectiveness in releasing biologics and drugs [188]. Additionally, biomaterials can support axonal growth, as aligned neurofibers have been shown to enhance neurite growth and cell adhesion in vitro, highlighting their potential for SCI treatment. However, surgically inserting preformed scaffolds poses challenges due to the nature of most SCIs. Injectable biomaterials could address this issue, but they must be deformable, requiring further research and development [188].

While all of these individual therapies have some drawbacks and contrasting evidence regarding their effectiveness in isolation, there is exciting research showing that when these interventions are used together, improved outcomes in SCI patients can follow. For example, a study in 2010 analyzed the combination of NPrCs, growth factors, and chondroitinase on SCI. Chondroitinase is an enzyme that inhibits the activity of CSPGs, thus facilitating long-term survival of transplanted NPrCs; specifically, the researchers found greater axonal density in the corticospinal tract after this therapy compared to controls [243]. Indeed, other preclinical trials have shown the effectiveness of combining chondroitinase with other molecular agents like anti-Nogo-A antibody and neurotrophin-3 in promoting better growth and plasticity in animal models with SCI [244]. Other studies have demonstrated the effectiveness of combining stem cells with molecular agents. For example, it seems that the pairing of methylprednisolone and mesenchymal stem cells can lead to decreased ROS formation, pro-inflammatory cytokine release, and apoptosis [244].

Another study found that neural stem cells injection combined with aerobic training increased stem cell differentiation and more central pattern generator activity in the mice that received both therapies compared to controls [247]. Along the same vein, a study from 2022 combined the injection of chondroitinase with epidural cortical electrical stimulation and behavioral rehabilitation. They found that when all these therapies were combined in mice, there was greater upper limb dexterity and muscle activity than in the control groups [248]. A study from 2013 demonstrated that exogenous BDNF administered in parallel with a reaching task in rats resulted in improved forelimb motor performance [245]. An additional study found that when a regimen of chondroitinase, growth factors, and daily treadmill training were combined in rats, there was more plasticity of the corticospinal tract and serotonergic–spinal pathway [246]. Although these preclinical trials do show promise in combinatorial therapies, further research must delineate which combinations confer synergy and undergo clinical trials.

## 7. Conclusions

Remyelination after SCI remains a critical therapeutic goal, and a growing body of preclinical and translational research has identified many promising molecular targets for intervention. The modulation of signaling pathways, administration of exogenous growth factors, pharmacological inhibitory interventions, and stem cell-based therapies all show encouraging results in animal models. However, the path to clinical translation is still impeded by the relative complexity and heterogeneity of human SCI and inconsistent functional recovery in human trials. A major unresolved barrier is the microenvironment of chronic inflammation, scarring, which impairs OPC proliferation and axonal stability. Furthermore, the crosstalk between the plethora of signaling cascades involved is not fully understood, thus making them difficult to reliably modulate via treatment.

As the field progresses, combinatorial strategies that integrate anti-inflammatory, regenerative, and rehabilitative modalities are likely to yield the most robust functional improvements. Physical rehabilitation, magnetic stimulation, and scaffold-based delivery platforms may enhance the efficacy of cell- and drug-based approaches by creating a permissive environment for remyelination and axonal regrowth. Based on our evaluation of the field, future progress and translational success will rely on tailoring therapies to the biological stage of injury and integrating multiple approaches to overcome barriers to remyelination.

## Figures and Tables

**Table 1:** A selection of ongoing clinical trials of SCI therapies as of 6 June 2025. Enrollment totals are actual. Data in this table was located using Clinicaltrials.gov [153] or the corresponding references listed in the table. References in the rightmost column were only provided if clinical trial results have been published.

Mechanism	Drug Name	Phase	Inclusion Criteria	Recruitment Status	Enrollment	Sponsor	Clinical Trial Identifier/Reference
Anti-Nogo-A antibodies	ATI355	1	C5-T12, paraplegic and tetraplegic patients with AIS A classification, 4–14 days post injury	Completed	52	Novartis Pharmaceuticals	NCT00406016/Kucher at al., 2018 [154]
Anti-Nogo-A antibodies	NG-101	2	C1-C8, tetraplegic patients with ASIA A-D classification; predicted UEMS recovery less than 41/50, 4–28 days post injury	Completed	129	University of Zurich	NCT03935321/Weidner et al., 2025 [155]
ROCK inhibitor	VX-210	2b/3	C4-C7, AIS A-B, within 72 h post injury	Completed	70	Vertex Pharmaceuticals Incorporated	NCT02669849
Tetracycline	Minocycline	1/2	C0-T11, motor complete or incomplete, within 12 h post injury	Completed	52	University of Calgary	NCT00559494/Casha et al., 2012 [156]
Inhibitor of lipid peroxidation	Tirilazad	3	Within 8 h post injury	Completed	499	National Institute of Neurological Disorders and Stroke (NINDS)	NCT00004759/Bracken et al., 1997 [157]
Hepatocyte Growth Factor	KP-100IT	3	C3-C8, AIS A, within 78 h post injury	Unknown Status	25	Kringle Pharma, Inc.	NCT04475224
FGF-1	SC0806	1/2	T2-T11, ASIA A, 4 months-10 years post injury	Unknown Status	27	BioArctic AB	NCT02490501

**Table 2:** An overview of the major therapeutic classes with their proposed mechanisms, levels of clinical evidence, and limitations.

Therapeutic Class	Mechanism	Evidence	Limitations/Challenges	References
Anti-Nogo-A antibodies (e.g., NG-101, ATI355)	Neutralize Nogo-A to reduce axonal growth inhibition and promote remyelination	Phase 1–2 trials completed	Efficacy is time- and region-specific; unclear translation to chronic SCI	[115,116,158,159,160,224,225,226,227,228,229,230,231,232,233,234,235,236,237,238,239,240,241,242,243,244]
ROCK inhibitors (e.g., VX-210)	Inhibit Rho/ROCK to reduce neuroinflammation and cytoskeletal collapse	Phase 2b/3 trial completed	Potential systemic side effects; dosing optimization needed	[125,161,162,224]
Shh agonists	Activate Shh pathway to drive OPC proliferation and differentiation	Preclinical rodent models	Delivery, specificity, and clinical translation remain uncertain	[72,73,181,182,183,227]
Notch inhibitors	Block Notch to permit OPC differentiation into OLs	Preclinical studies in SCI and MS	Broad effects on Notch signaling may have off-target risks	[63,64,66,224]
Wnt/β-catenin inhibitors (e.g., XAV939)	Suppress Wnt signaling to enable OPC maturation and reduce glial scarring	Preclinical in vitro and in vivo models	Timing-sensitive; may interfere with development or repair if over-suppressed	[62,85,184,191,224]
Minocycline	Anti-inflammatory, reduces apoptosis and microglial activation	Phase 1–2 clinical trial completed	Clinical benefit unclear; variability in outcomes and dosage requirements	[125,156,166,224]
HDAC inhibitors (e.g., CI-994, VPA)	Increase myelin gene transcription via chromatin remodeling	Preclinical models of SCI and demyelination	Possible off-target transcriptional effects; needs controlled delivery	[109,111,175,177,224]
Growth factor delivery (e.g., BDNF, IGF-1, FGF)	Enhance OL survival and differentiation via trophic support	Preclinical studies; early human biomaterial trials ongoing	Growth factor degradation, short half-life, and targeting challenges	[58,65,76,77,78,79,91,116,158,193,195,196,200,207,209,224,243,245,246]
Sodium channel blockers (e.g., Riluzole, Phenytoin)	Reduce excitotoxicity and OL damage by blocking Na+ influx	Preclinical SCI models	Nonspecific action; potential motor side effects and seizure risk	[188,189,190,192,224]
Antioxidants (e.g., Edaravone)	Scavenge ROS to protect OLs and support remyelination	Rodent models of SCI and white matter damage	Limited CNS penetration; unclear clinical efficacy beyond ALS	[147,148]
Cell therapy (OPCs, iPSC-derived NSCs, MSCs)	Replace or augment endogenous OPCs; modulate immune microenvironment	Multiple preclinical models; early-phase human trials	Low survival/integration; immune rejection risks; scalability issues	[163,164,165,166,167,168]
Gene therapy (e.g., CRISPR-edited OPCs)	Improve OPC function or lesion resistance via gene editing (e.g., Daam2 KO)	Rodent models with functional and histological improvement	Ethical and regulatory hurdles; specificity of edits and durability	[156]
MSC or OPC-derived EVs	Deliver pro-regenerative miRNAs or proteins to injury site	Preclinical models	Standardization, biodistribution, and scaling remain barriers	[172,173,174]
Neurostimulation (TMS, FES, treadmill training)	Stimulate neurotrophic signaling and OL maturation through activity-dependent mechanisms	Human and rodent studies; some meta-analyses available	Inconsistent functional outcomes; requires intensive rehabilitation	[179,180,181,182,183,184,185,186,187]

## Abbreviations

The following abbreviations are used in this manuscript:

SCI	Spinal cord injury
OPC	Oligodendrocyte progenitor cell
OL	Oligodendrocyte
CNS	Central nervous system
GPCR	G protein-coupled receptor
ROS	Reactive oxygen species
MS	Multiple sclerosis
Shh	Sonic hedgehog
BDNF	Brain-derived neurotrophic factor
IGF-1	Insulin-like growth factor
FGF	Fibroblast growth factor
MBP	Myelin basic protein
SC	Spinal cord
RNS	Reactive nitrogen species
TNF-α	Tumor necrosis factor-α
MAI	Myelin-associated inhibitor
MAG	Myelin-associated glycoprotein
OMgp	Oligodendrocyte myelin glycoprotein
BMP	Bone morphogenetic protein
CSPG	Chondroitin sulfate proteoglycans
RGMA	Repulsive guidance molecule A
ROCK	Rho-associated kinase
FES	Functional electrical stimulation
NPrC	Neuronal precursor cell
sNPC	Spinal neuronal progenitor cell
TMS	Transcranial magnetic stimulation
Gap43	Growth-associated protein 43
AKT	Protein kinase B
PI3K	Phosphoinositide 3-kinase
cAMP	Cyclic adenosine monophosphate
PKA	Protein kinase A
EPAC	Exchange protein directly activated by cAMP
CREB	cAMP response element-binding protein
PDE	Phosphodiesterase
Id	Inhibitors of differentiation
ID	Inhibitor of DNA binding
bHLH	Basic helix-loop-helix
AIS	ASIA impairment scale
CPG	Central pattern generator
NPC	Neural progenitor cell
MSC	Mesenchymal stromal/stem cell
HDAC	Histone deacetylase
DNMT	DNA methyltransferase
siRNA	Small interfering RNA
iPSC	Induced pluripotent stem cell
EV	Extracellular vesicle
Nrf2	Nuclear erythroid 2-related factor
GPX4	Glutathione peroxidase 4
NF-κB	Nuclear factor kappa-B
